# Biochemical, urinary, and acid-base profile in cattle treated with maintenance enteral electrolyte solutions containing calcium propionate, propylene glycol or glycerol

**DOI:** 10.3389/fvets.2022.945542

**Published:** 2022-09-08

**Authors:** Pedro Ancelmo Nunes Ermita, Rinaldo Batista Viana, Marcel Ferreira Bastos Avanza, Raffaela Bertoni Cavalcanti Teixeira, José Ricardo Barboza Silva, Lorena Chaves Monteiro, Caio Monteiro Costa, Lucas Drumond Bento, Paulo Vinicius da Costa Mendes, Dayana Alersa Conceição Ferreira Ermita, Brenda Ventura Lopes Carvalho, Nadyne Souza Moreira, Maria Carolina Neves de Souza, José Dantas Ribeiro Filho

**Affiliations:** ^1^Institute of Humid Tropic Studies, Federal University of South and Southeast of Pará, Xinguara, Brazil; ^2^Institute of Animal Health and Production, Federal Rural University of the Amazon, Belém, Brazil; ^3^Laboratory of Research in Veterinary Internal Medicine, Veterinary Department, Viçosa Federal University, Viçosa, Brazil

**Keywords:** fluid therapy, continuous flow, naso-ruminal tube, energy sources, laboratorial analysis

## Abstract

Enteral fluid therapy administered in continuous flow through the naso-ruminal route for long periods with electrolyte solutions is safe and effective in cattle. The aim of this study was to carry out a comparative assessment between maintenance enteral electrolyte solutions containing calcium propionate, propylene glycol or glycerol administered in continuous flow in cattle. Six heifers were used and the study was carried out in a 6 × 3 crossover design, in which each animal received three different treatments: enteral electrolyte solution containing calcium propionate (ESCaP), enteral electrolyte solution containing glycerol (ESGly) and enteral electrolyte solution containing propylene glycol (ESPrG). Solutions were administered at a rate of 15 mL kg^−1^ h^−1^ for 12 h. Serum and urinary biochemical assessment; urinary volume, pH, and specific gravity; and blood gas analysis were measured at 0, 3, 6, 9, 12, and 24 h. All three enteral electrolyte solutions expanded blood volume and increased urine volume without causing electrolyte imbalances. ESCaP caused mild reversible metabolic alkalosis while the most significant glycemic potential was observed in electrolyte solutions containing propylene glycol (ESPrG) and calcium propionate (ESCaP).

## Introduction

Enteral fluid therapy (EFT) has been classically used to correct hydroelectrolytic and acid-base imbalances in adult ruminants due to its effectiveness and practicality. This therapeutic modality allows the administration of large volumes of fluids directly into the rumen, which will be absorbed through an osmotic gradient established between the rumen contents and the blood plasma (1). In this modality, the electrolyte solution is infused through the oro-ruminal route as a bolus.

The enteral fluid therapy in continuous flow (EFTcf) is performed through naso-ruminal route using a small-caliber tube and allows the electrolyte solution (ES) to be administered slowly. It reduces animals' stress because it minimizes rumen distension in comparison to the oro-ruminal bolus infusion route, in which large volumes are administered in successive passages of oro-ruminal tube. Furthermore, EFTcf allows the patient to move freely and eat during therapy without compromising its performance and therapeutic efficacy (2).

Maintenance enteral electrolyte solutions must be able to replace water and electrolytes without causing new imbalances, even when used for long periods (3). Therefore, the composition of the solution has central importance in the success of the therapy. The ideal formulation for adult cattle remains unknown, especially when it is necessary to use energy precursors. It is suggested that enteral electrolyte solution must contain sodium, potassium, chloride, calcium, magnesium, and phosphorus (4). In addition, the solution should not cause any adverse effects. As inappetence is a frequent finding in diseases, it is also widely recommended that an energy source should be added to the ES (5).

In adult cattle, due to ruminal activity, it is recommended to use gluconeogenic precursors such as propionate, glycerol, and propylene glycol. These compounds can be absorbed by the ruminal epithelium or undergo fermentation and are then converted into propionate and used in energy production through gluconeogenesis (6).

The present study aimed to evaluate three enteral electrolyte solutions administered *via* naso-ruminal route in continuous flow during maintenance therapy containing calcium propionate, glycerol or propylene glycol and their effects on biochemical, urinary, and acid-base profile of cattle. Our hypothesis was that EFTcf with enteral electrolyte solutions containing gluconeogenic precursors are effective in increasing glycemic rate and blood volume in cattle without causing adverse effects.

## Materials and methods

### Experimental design

This study was a controlled trial in a cross-over design. The animals were randomly assigned to a 6 × 3 crossover design (six animals × three treatments). All animals were submitted to all treatments, which were applied under the same conditions, with seven days interval to avoid the overlap of effects. Six healthy Holstein heifers, aged between 18 and 24 months and average body weight of 300 ± 25 kg, from the Experimental Unit for Research and Extension in Dairy Cattle at the Federal University of Viçosa were used. The heifers were considered healthy based on clinical and laboratory tests. The animals were placed in individual pens in a tie stall system 10 days prior to the beginning of the experiment to get acclimatized to the environment. The feeding was based on corn silage, balanced ration, water, and mineral supplementation *ad libitum*, according to the management adopted in the unit.

The effects of three treatment solutions with the following compositions were evaluated: Enteral electrolyte solution containing calcium propionate (ESCaP) - 4 g of sodium chloride (Sulfal Química, Brazil), 0.5 g of potassium chloride (Sulfal Química, Brazil), 0.3 g of magnesium chloride (Sulfal Química, Brazil) and 10 g of calcium propionate (Adicel, Brazil) for 1,000 mL of solution (measured osmolarity: 299 mOsm/L); Enteral electrolyte solution containing glycerol (ESGly) - 4 g of sodium chloride (Sulfal Química, Brazil), 0.5 g of potassium chloride (Sulfal Química, Brazil), 0.3 g of magnesium chloride (Sulfal Química, Brazil), 1 g of calcium acetate (Sulfal Química, Brazil) and 10 mL of glycerol (BioBrotas Olequímica, Brazil) to 1,000 mL of solution (measured osmolarity: 287 mOsm/L); Enteral electrolyte solution containing propylene glycol (ESPrG) – 4 g of sodium chloride (Sulfal Química, Brazil), 0.5 g of potassium chloride (Sulfal Química, Brazil), 0.3 g of magnesium chloride (Sulfal Química, Brazil), 1 g of calcium acetate (Sulfal Química, Brazil) and 15 mL of propylene glycol (Sulfal Química, Brazil) to 1,000 mL of solution (measured osmolarity: 378 mOsm/L).

A naso-ruminal tube with 1 cm in diameter and 1.8 m in length was used to stablish enteral fluid therapy in continuous flow. Before starting treatments, the tube was introduced into one of the nostrils and attached to the halter of each animal. The animals were kept in individual pens in a tie stall system throughout the experimental period. The solutions were administered for 12 h in a continuous flow rate of 15 mL kg^−1^ h^−1^, which was based on our clinical routine and veterinary medicine clinical trials (7–9).

Laboratory evaluations were performed at the following times: T0h – immediately before fluid therapy; T3h – 3 h of fluid therapy; T6h – 6 h of fluid therapy; T9h – 9 h of fluid therapy; T12h – 12 h of fluid therapy; and T24h – 24 h after fluid therapy. Urine samples were obtained in all time points except for the last one (T24h). The animals did not have access to water and food during the 24 h of the experimental trial.

### Clinical evaluations and collection of biological samples

Blood samples were collected by jugular venipuncture in tubes (Labor Import, People's Republic of China) containing sodium fluoride/EDTA to obtain plasma, and without anticoagulant to obtain serum, using a vacuum collection system (BD Vacutainer, Brazil). Venous blood samples for blood gas analysis were obtained using plastic syringes containing lithium heparin. Urine samples were obtained directly from the urinary bladder, through a number 32 Foley catheter, which was introduced into the urethra and had the cuff filled with sterile saline solution. A plastic hose was connected to the urinary catheter and attached to a bucket to collect all the urine produced.

Serum biochemical analysis of sodium and potassium were performed by flame photometry (Fotometro B462 Micronal, Brazil). The analysis of chloride, total calcium, magnesium, glucose, lactate, total serum proteins, urea and creatinine were performed in an automatic HumaStar 300 (InVitro Diagnóstica, Brazil) using commercial kits (BioClin Quibasa, Brazil) and according to the manufacturer's recommendations. Serum osmolarity was determined by the freezing point depression technique in a model 3320 osmometer (Advanced Instruments, USA). Blood gas analysis was performed immediately after blood collection, in an OMNI C blood gasometer (Roche Diagnóstica, Brazil) and included the following parameters: pH, partial pressure of carbon dioxide (pCO_2_), bicarbonate concentration (HCO${}_{3}^{-}$), and base excess (BE).

### Calculations

The determination of Anion Gap (AG) and Strong Ion Difference (SID) values were performed using the serum concentrations of strong ions and according to the following equations (10):


$$\begin{array}{l} \text{AG }(\text{mmol/L})\text{ = }({\text{Na}}^{\text{+}}\text{ + }{\text{K}}^{\text{+}})\text{ – }({\text{Cl}}^{\text{–}}{\text{ + HCO}}_{\text{3}}^{\text{–}})\\ \text{SID }(\text{mmol/L})\text{ = }[({\text{Na}}^{\text{+}}{\text{ + K}}^{\text{+}})\text{ – }({\text{Cl}}^{\text{–}}) \end{array}$$

Urinary sodium, potassium, and chloride were analyzed in an automatic HumaStar 300 (InVitro Diagnóstica, Brazil) using commercial kits (BioClin Quibasa, Brazil) and according to the manufacturer's recommendations. Urine specific gravity (Refractometer), urinary volume in liters, and urinary pH (pH Metro Digital – Del Lab, Brazil) were determined.

### Statistical analysis

Data were submitted to descriptive analysis and the results presented in tables using mean and standard deviation. The normality of the data distribution and the sphericity of the variances were evaluated using the Shapiro-Wilk and Mauchly tests, respectively. ANOVA was used based on a factorial design of repeated measures to evaluate the effects of time, treatments, and the time ^*^ treatment interaction. To perform the multiple comparisons, Fisher's least significant difference test was used. When it was not possible to use ANOVA, the non-parametric Friedman test with Wilcoxon *post hoc* associated with Bonferroni correction was used. All analyzes were performed using the SPSS statistical software version 20 (IBM, USA). Significance was considered when *P* < 0.05.

## Results

There were no changes (*P* > 0.05) in the serum concentrations of sodium, potassium, chloride, total calcium, magnesium, and urea as a function of time and treatment used (Table 1). There was a significant reduction in creatinine values during the fluid therapy phase in the ESCaP group between T0h and T12h and in the ESGly group between T3h and T12h (Table 1).

**Table 1:** Mean ± standard deviation of serum electrolytes sodium (Na^+^), potassium (K^+^), chloride (Cl^−^), total calcium (tCa^++^) magnesium (Mg^++^), urea, and creatinine in cattle submitted to continuous flow enteral fluid therapy with three electrolyte solutions containing different energy precursors.

**Variable**	**Fluid therapy period**						**Post Fluid** **Therapy**
	**Trat^1^**	**T0h**	**T3h**	**T6h**	**T9h**	**T12h**	**T24h**
Na^+^ mMol/L	ESCaP	133,7 ± 1,2	130,0 ± 5,0	137,3 ± 7,4	131,4 ± 11,0	131,5 ± 4,4	131,0 ± 8,8
	ESGly	135,0 ± 10,7	140,8 ± 12,9	137,6 ± 5,7	128,7 ± 15,6	130,5 ± 3,4	136,0 ± 8,2
	ESPrG	127,5 ± 3,0	132,8 ± 3,4	132,3 ± 11,2	135,0 ± 10,0	144,0 ± 10,6	138,0 ± 8,9
K^+^ mMol/L	ESCaP	4,5 ± 1,2	4,4 ± 1,6	4,0 ± 0,4	3,7 ± 0,6	3,9 ± 0,7	4,1 ± 0,9
	ESGly	4,7 ± 0,9	4,5 ± 1,1	4,1 ± 1,2	3,3 ± 0,8	3,6 ± 0,4	3,5 ± 0,6
	ESPrG	4,4 ± 1,2	3,9 ± 0,4	3,9 ± 0,7	4,0 ± 0,5	4,4 ± 0,8	3,9 ± 0,6
Cl^−^ mMol/L	ESCaP	95,0 ± 2,2	95,8 ± 1,0	94,9 ± 3,5	94,0 ± 4,8	96,7 ± 2,3	94,2 ± 3,9
	ESGly	94,5 ± 3,8	96,7 ± 1,9	96,2 ± 0,7	96,9 ± 0,8	96,3 ± 2,3	98,4 ± 1,2
	ESPrG	96,0 ± 5,1	95,0 ± 1,6	95,4 ± 3,9	96,9 ± 2,0	97,2 ± 2,2	94,7 ± 1,4
tCa^++^ m**g**/**d**L	ESCaP	12,6 ± 1,4	13,0 ± 0,4	12,2 ± 1,6	11,8 ± 2,5	13,5 ± 1,5	12,9 ± 1,1
	ESGly	12,8 ± 1,5	12,7 ± 1,0	13,0 ± 0,6	13,1 ± 1,0	11,0 ± 5,5	13,9 ± 0,6
	ESPrG	13,8 ± 1,8	13,0 ± 0,6	13,0 ± 0,9	13,1 ± 0,5	13,0 ± 0,9	13,8 ± 0,8
Mg^++^ mg/dL	ESCaP	2,1 ± 0,2	2,3 ± 0,6	2,0 ± 0,3	1,8 ± 0,4	1,8 ± 0,2	1,8 ± 0,3
	ESGly	2,3 ± 0,6	2,2 ± 0,2	1,9 ± 0,2	2,0 ± 0,2	2,0 ± 0,2	1,9 ± 0,2
	ESPrG	2,2 ± 0,3	2,0 ± 0,1	2,1 ± 0,1	2,0 ± 1,0	2,1 ± 0,1	2,1 ± 0,1
Urea mg/dL	ESCPa	6,3 ± 4,2	8,8 ± 2,0	7,2 ± 3,7	8,3 ± 2,5	7,7 ± 3,8	11,0 ± 3,1
	ESGly	9,1 ± 5,0	7,2 ± 3,1	8,5 ± 2,5	6,8 ± 1,5	6,1 ± 2,8	7,1 ± 1,5
	ESPrG	8,0 ± 3,8	8,8 ± 2,7	9,5 ± 3,2	9,9 ± 2,8	8,4 ± 2,2	10,8 ± 3,3
Creatinine mg/dL	ESCaP	0,6 ± 0,2^a^	0,5 ± 0,1^bc^	0,5 ± 0,1^bc^	0,6 ± 0,1^ad^	0,5 ± 0,1^bd^	0,6 ± 0,1^ac^
	ESGly	0,6 ± 0,2^ab^	0,6 ± 0,1^a^	0,6 ± 0,1^ab^	0,6 ± 0,1^a^	0,5 ± 0,1^b^	0,6 ± 0,1^a^
	ESPrG	0,6 ± 0,2	0,6 ± 0,2	0,6 ± 0,1	0,6 ± 0,1	0,6 ± 0,2	0,6 ± 0,1

Means followed by different lowercase letters in the same row and different uppercase letters in the same column differ by α = 5%. Trat^1^ = Treatment: ESCaP = electrolyte solution with calcium propionate; ESGly = electrolyte solution with glycerol; ESPrG = electrolyte solution with propylene glycol.

There was a significant reduction in total serum protein values (Table 2) between T0h and T9h in ESCaP treatment, and between T0h and T6h in ESGly and ESPrG treatments. However, at the end of the fluid therapy phase (T12h) all values returned to baseline (T0h). Serum osmolarity (Table 2) decreased (*P* < 0.05) in ESCaP treatment between T0h and T3h and returned to baseline values at T6h. In the ESGly treatment no statistical difference between the timepoints in relation to T0h was observed. An increase in osmolarity (*P* < 0.05) between T3h and T6h was noted. In the ESPrG treatment group a significant increase in serum osmolarity was observed at the end of the fluid therapy phase (T12h) when compared to T0h values.

**Table 2:** Mean ± standard deviation of total serum protein (TSP), serum osmolarity (Osm), glucose and lactate in cattle submitted to continuous flow enteral fluid therapy with three electrolyte solutions containing different energy precursors.

**Variable**	**Fluid therapy period**						**Post fluid** **therapy**
	**Trat^1^**	**T0h**	**T3h**	**T6h**	**T9h**	**T12h**	**T24h**
TSP g/dL	ESCaP	7,2 ± 0,6ª	7,4 ± 0,9^ab^	7,1 ± 0,7^ab^	6,9 ± 0,5^b^	7,4 ± 0,6^ab^	7,5 ± 0,3^a^
	ESGly	7,3 ± 0,7^ac^	6,9 ± 0,6^abc^	6,8 ± 0,7^b^	7,0 ± 0,9^ab^	7,5 ± 0,9^abc^	7,4 ± 0,9^c^
	ESPrG	7,5 ± 0,8^a^	7,6 ± 0,8^a^	7,0 ± 0,7^b^	7,0 ± 0,6^ab^	7,1 ± 0,7^ab^	7,2 ± 0,7^ab^
Osm mOsm/L	ESCaP	284,3 ± 9,1^a^	280,3 ± 3,3^b^	288,5 ± 4,8^ab^	288,2 ± 10,7^ab^	293,3 ± 5,7^a^	290,8 ± 7,2^a^
	ESGly	282,3 ± 7,8^ab^	279,8 ± 4,2^b^	284,3 ± 3,9^a^	284,3 ± 4,6^ab^	293,5 ± 13,2^ab^	286,5 ± 3,3^a^
	ESPrG	284,0 ± 5,4^b^	283,2 ± 4,4^b^	288,0 ± 5,7^b^	293,2 ± 5,9^ab^	298,7 ± 11,0^a^	284,4 ± 4,5^ab^
Glucose mMol/L	ESCaP	4,6 ± 0,2^a^	4,7 ± 0,2^ab^	4,9 ± 02^b^	5,0 ± 0,4^ab^	4,8 ± 0,1^b^	4,1 ± 0,2^c^
	ESGly	4,6 ± 0,3	4,7 ± 0,4	4,7 ± 0,5	4,7 ± 0,6	4,7 ± 0,3	4,6 ± 0,3
	ESPrG	4,6 ± 0,3^c^	4,9 ± 0,3^ab^	5,0 ± 0,3^ab^	5,0 ± 0,4^a^	4,8 ± 0,3^b^	4,7 ± 0,4^abc^
Lactate mMol/L	ESCaP	1,0 ± 0,7	0,4 ± 0,1	0,4 ± 0,1	0,5 ± 0,1	0,4 ± 0,2	0,8 ± 0,3
	ESGly	0,6 ± 0,1	0,5 ± 0,1	0,4 ± 0,1	0,4 ± 0,1	0,4 ± 0,1	0,7 ± 0,3
	ESPrG	0,7 ± 0,5	8,0 ± 0,8	0,6 ± 0,1	0,5 ± 0,1	0,6 ± 0,2	0,6 ± 0,2

Means followed by different lowercase letters in the same row and different uppercase letters in the same column differ by α = 5%. Trat^1^ = Treatment: ESCaP = electrolyte solution with calcium propionate; ESGly = electrolyte solution with glycerol; ESPrG = electrolyte solution with propylene glycol.

Plasma glucose increased (*P* < 0.05) in ESCaP between T0h and T6h and remained high until T12h, but at the end of the trial (T24h) blood glucose was lower than T0h. There was no glycemic change in ESGly (Table 2). In ESPrG plasma glucose increased significantly in the first h of fluid therapy (T3h) and remained high until T12h, returning to initial values at T24h. There was no effect (*P* > 0.05) of time or treatments over time on plasma lactate concentration.

Urinary sodium increased over time in the animals of all treatments, reaching the highest values at T12h. In turn, urinary potassium values decreased in animals from all treatments, reaching the lowest values at T12h. Urinary chloride remained unchanged over time in the ESGly, while in animals that received ESCap and ESPrG, sligth increases were recorded at T12h (Table 3).

**Table 3:** Mean ± standard deviation of urinary electrolytes (urNa^+^, urK^+^, urCl^−^), urine specific gravity, and urinary volume in cattle submitted to continuous flow enteral fluid therapy with three electrolyte solutions containing different energy precursors.

**Variable**	**Fluid therapy period**					
	**Trat^1^**	**T0h**	**T3h**	**T6h**	**T9h**	**T12h**
urNa^+^ mMol/L	ESCaP	7,3 ± 3,0^bc^	26,2 ± 26,2^b^	33,3 ± 16,2^c^	69,3 ± 16,7^a^	79,8 ± 15,1^a^
	ESGly	8,8 ± 3,5^b^	22,5 ± 24,9^ab^	36,2 ± 21,7^ab^	57,3 ± 22,7^a^	66,2 ± 17,4^a^
	ESPrG	14,0 ± 14,3^b^	11,8 ± 4,8^bc^	19,0 ± 9,9^bc^	46,0 ± 20,7^ac^	54,2 ± 12,8^a^
urK^+^ mMol/L	ESCaP	57,2 ± 45,0	34,5 ± 23,9	36,0 ± 24,8	23,8 ± 7,5	19,3 ± 5,9
	ESGly	78,0 ± 42,7^a^	60,7 ± 42,4^ab^	26,8 ± 7,2^a^	16,2 ± 2,2^bc^	13,5 ± 7,2^c^
	ESPrG	95,8 ± 77,4^a^	51,0 ± 35,9^a^	22,0 ± 15,5^ab^	25,3 ± 14,5^ab^	18,5 ± 8,8^b^
urCl^−^ mMol/L	ESCaP	45,6 ± 29,1^ab^	42,2 ± 19,4^b^	63,3 ± 14,3^ab^	73,5 ± 8,0^a^	75,7 ± 8,6^a^
	ESGly	65,0 ± 29,2	67,7 ± 34,3	61,3 ± 14,5	70,6 ± 6,1	66,9 ± 13,0
	ESPrG	62,7 ± 29,4^ab^	62,7 ± 37,2^ab^	39,0 ± 16,2^b^	61,2 ± 17,1^a^	68,2 ± 18,9^a^
Urine specific gravity	ESCaP	1,014 ± 11^ab^	1,006 ± 4^ab^	1,004 ± 3^ab^	1,003 ± 2^a^	1,004 ± 2^b^
	ESGly	1,015 ± 12	1,012 ± 11	1,003 ± 2	1,004 ± 1	1,003 ± 1
	ESPrG	1,016 ± 14^a^	1,010 ± 8^a^	1002 ± 1^b^	1004 ± 1^ab^	1,005 ± 3^ab^
Urinary volume (mL)	ESCaP	-	2,167 ± 973^b^	4,633 ± 1847^c^	7,817 ± 3326^a^	8,375 ± 2236^a^
	ESGly	-	2,308 ± 1175^b^	5,092 ± 1608^c^	8,075 ± 2926^d^	12,067 ± 4001^a^
	ESPrG	-	1,817 ± 1610^b^	4,275 ± 1329^b^	5,617 ± 2481^b^	9,367 ± 3619^a^

Means followed by different lowercase letters in the same row and different uppercase letters in the same column differ by α = 5%. Trat^1^ = Treatment: ESCaP = electrolyte solution with calcium propionate; ESGly = electrolyte solution with glycerol; ESPrG = electrolyte solution with propylene glycol.

Urine specific gravity (Table 3) did not change in the ESCaP and ESGly groups in relation to the initial values (T0h). A significant decrease was observed only in ESPrG group (*P* < 0.05) between T0h and T6h. Urinary volume (Table 3) progressively increased up to T9h in the ESCaP group and up to T12h in the ESGly group. In the ESPrG group a significant increase in urinary volume was observed only at T12h.

Blood pH values (Table 4) increased (*P* < 0.05) in the ESCaP group at T9h and remained high until the end of fluid therapy (T12h). In the ESGly group there was a significant increase between T3h and T9h, that extended until T12h. In both groups values returned to baseline conditions at T24h. There was no effect of time or treatments on blood pH in the ESPrG group. The pCO_2_ values (Table 4) remained unchanged (*P* > 0.05) in all treatments, while the HCO${}_{3}^{-}$ concentration increased significantly in all treatments at T9h in relation to T0h and remained high at T12h. HCO${}_{3}^{-}$ concentration returned to baseline at T24h.

**Table 4:** Mean ± standard deviation of the blood gas parameters, anion gap (AG), strong ion difference (SID), and urinary pH in cattle submitted to continuous flow enteral fluid therapy with three electrolyte solutions containing different energy precursors.

**Variable**	**Fluid therapy period**						**Post fluid** **therapy**
	**Trat^1^**	**T0h**	**T3h**	**T6h**	**T9h**	**T12h**	**T24h**
pH	ESCaP	7,40 ± 0,03^b^	7,39 ± 0,01^b^	7,40 ± 0,02^b^	7,42 ± 0,02^a^	7,44 ± 0,04^a^	7,41 ± 0,02^ab^
	ESGly	7,39 ± 0,03^ab^	7,38 ± 0,03^b^	7,40 ± 0,02^b^	7,41 ± 0,03^a^	7,41 ± 0,03^a^	7,40 ± 0,03^ab^
	ESPrG	7,40 ± 0,02^a^	7,38 ± 0,06^a^	7,39 ± 0,02^a^	7,40 ± 0,01^a^	7,39 ± 0,03^a^	7,39 ± 0,03^a^
pCO_2_ mmHg	ESCaP	48,8 ± 4,4	48,9 ± 4,7	51,0 ± 3,1	50,6 ± 4,1	51,4 ± 5,2	50,4 ± 6,4
	ESGly	48,7 ± 3,4	48,6 ± 2,8	49,2 ± 4,3	49,6 ± 2,8	50,2 ± 3,5	47,8 ± 3,2
	ESPrG	47,3 ± 3,5	50,9 ± 9,3	48,2 ± 2,5	48,6 ± 4,6	51,7 ± 1,8	51,2 ± 6,3
HCO${}_{3}^{-}$ mMol/L	ESCaP	29,7 ± 3,2^bd^	29,1 ± 2,7^b^	30,8 ± 1,5^bc^	32,5 ± 3,2^ac^	33,9 ± 1,7^a^	32,1 ± 2,2^acd^
	ESGly	29,1 ± 2,4^b^	29,0 ± 2,2^b^	29,5 ± 2,8^ab^	30,9 ± 2,3^a^	30,9 ± 2,4^a^	28,6 ± 1,4^b^
	ESPrG	28,3 ± 1,1^b^	29,4 ± 1,4^ab^	28,7 ± 2,2^ab^	30,8 ± 1,7^a^	30,9 ± 1,3^a^	30,4 ± 2,5^ab^
BE mMol/L	ESCaP	3,9 ± 3,0^bc^	3,2 ± 2,2^b^	4,9 ± 1,4^bcd^	6,7 ± 2,9^ad^	8,1 ± 1,5^a^	6,0 ± 1,9^cd^
	ESGly	3,2 ± 2,4^b^	3,2 ± 2,1^b^	3,8 ± 2,6^abc^	5,1 ± 2,3^a^	5,0 ± 2,3^ac^	2,9 ± 1,5^bc^
	ESPrG	2,9 ± 1,3^a^	3,4 ± 2,0^a^	3,2 ± 2,3^a^	4,9 ± 1,6^a^	4,7 ± 1,7^a^	4,2 ± 1,9^a^
AG mMol/L	ESCaP	13,6 ± 1,5	11,6 ± 2,9	16,8 ± 4,5	17,7 ± 10,3	5,0 ± 5,3	17,6 ± 3,7
	ESGly	18,0 ± 9,0	20,8 ± 11,7	20,8 ± 6,7	14,6 ± 5,4	9,9 ± 2,7	13,5 ± 8,7
	ESPrG	7,8 ± 3,3	12,6 ± 5,7	8,3 ± 10,2	12,2 ± 8,1	20,4 ± 11,2	16,3 ± 9,1
SID mMol/L	ESCaP	39,9 ± 7,2	39,0 ± 7,4	45,2 ± 1,6	36,9 ± 10,2	36,0 ± 18,0	44,0 ± 16,3
	ESGly	41,8 ± 12,0	37,9 ± 2,8	46,8 ± 7,1	37,7 ± 13,6	38,2 ± 5,4	45,2 ± 9,1
	ESPrG	36,1 ± 0,5	37,9 ± 11,7	39,9 ± 10,9	42,7 ± 17,4	40,1 ± 10,0	41,9 ± 14,1
Urinary pH	ESCaP	7,2 ± 0,8	7,5 ± 0,6	7,1 ± 0,9	7,4 ± 1,0	7,7 ± 0,9	-
	ESGly	7,5 ± 0,9	7,3 ± 0,8	7,2 ± 0,6	7,1 ± 0,9	7,2 ± 0,8	-
	ESPrG	7,5 ± 0,8	7,2 ± 0,8	7,1 ± 0,6	7,0 ± 0,7	7,3 ± 0,6	-

Means followed by different lowercase letters in the same row and different uppercase letters in the same column differ by α = 5%. pH, blood pH; pCO_2_, Carbon dioxide pressure; HCO${}_{3}^{-}$, bicarbonate concentration; BE, Base excess; AG, Anion Gap; SID, Strong ion difference.

In the animals of the ESCaP and ESGly groups, the base excess (BE) increased (*P* < 0.05) at T9h and T12h, when compared to T0h values. No changes in BE were observed in the ESPrG group (Table 4). The anion Gap (AG) and the strong ion difference (SID) showed no difference between groups (Table 4), nor in the treatments throughout the experimental phase. Urinary pH (Table 4) also remained unchanged between groups and within groups over time (*P* < 0.05).

## Discussion

The stability of serum sodium, potassium, and chloride concentrations, as observed in the present study, is a necessary condition during fluid maintenance therapy as it decreases the chances of iatrogenic occurrence of hydroelectrolytic and acid-base disorders, in addition to correcting existing imbalances (2). The maintenance of serum calcium levels in the animals of this study could be attributed to the calcium propionate and calcium acetate present in the ES. Therefore, these salts are good sources of calcium to be used during the maintenance phase of enteral fluid therapy in cattle. Interestingly, the same can be attributed to the stability of magnesium. Studies using enteral fluid therapy in large animals have reported a reduction in serum calcium (2) and serum magnesium (11, 12). In all cases those changes were attributed to a lack of a source of these electrolytes in the solutions.

The decrease in creatinine concentration observed in the ESCaP and ESGly groups was due to volume expansion, more marked in these groups, possibly because of the lower osmolarity of the solutions. According to Avanza et al. (12), electrolyte solutions with low osmolarity are absorbed in greater amounts by the gastrointestinal tract. Urea and creatinine concentrations, when evaluated together, have been used to assess renal function. Due to the particularities of urea metabolism in ruminants, this parameter does not have a good relationship with glomerular function (13). Thus, the composition of the diet offered to these animals might be responsible for urea and creatinine values below the reference limit for the species. According to Rennó et al. (14), urea values below the reference may be due to alterations in the crude protein: metabolizable energy ratio. Low creatinine values can also be found in animals with low muscle mass (15).

All solutions used were able to expand blood volume with a significant decrease in total seric protein, similar as described by Ribeiro Filho et al. (16) in adult cattle. It was also expected that serum osmolarity would decrease, but this was not observed in the present study. As cited by Carlson and Bruss (17), the main substances responsible for serum osmolarity are sodium, glucose and urea. In the animals of the present study, serum sodium and urea values were not altered, while slight increase in glucose value was observed in ESCaP and ESPrG treatments. This increase could explain the changes in serum osmolarity in these groups. Alves et al. (7) did not observe changes in serum osmolarity during fluid therapy in adult cows using the same protocol of the present study, however, their solutions did not have energy precursor in the composition.

Although glycemic levels at the beginning of the study were already high in all groups, it was still possible to observe an increase in blood glucose in the ESCaP and ESPrG groups, confirming the glycemic potential of calcium propionate and propylene glycol. The longer lasting effect of ESPrG is probably due to the higher osmolarity of the solution (378 mOsm/L), which makes it remain for a longer period in the intestinal lumen and to be more slowly absorbed. Propylene glycol is slowly metabolized in the rumen environment (18) and the main product of hepatic metabolism of this substance is lactate, which is used in gluconeogenesis (19). An increase in serum lactate could be expected, but it was not observed in the present study reinforcing the effectiveness of this precursor for maintenance fluid therapy.

It was observed a gradual increase on urinary sodium (urNa^+^) excretion during fluid therapy, achieving higher rate of excretion at T12h (end of fluid therapy). Besides that, serum hyponatremia was not detected. It is important to highlight that this increase, beyond the contribution of solutions' composition, had been influenced by the reduction of antidiuretic hormone and renin-angiotensin- aldosterone system. The blood volume expansion due to enteral fluid therapy results in the reduction of aldosterone concentration and, possibly, activation of other mechanisms of circulating blood volume control, such as release of natriuretic peptide. In this mechanism, the inhibition of sodium reabsorption at nephron collecting ducts determines higher urinary excretion of sodium. As mentioned by Ribeiro Filho et al. (20), it should be noted that the use of hypotonic electrolytic enteral solutions, mostly administered for long periods, should be constantly monitored, especially in animals with pronounced hyponatremia.

Aldosterone is the main modulator of potassium excretion in urine, by increasing the activity of the enzyme Na/K-ATPase in the basolateral membrane of the collecting ducts, increasing sodium absorption and potassium excretion (21). The inhibition of the aldosterone effects in the collecting ducts by the aforementioned mechanism justifies the reduced concentration of urK^+^ during enteral fluid therapy. These variations in urNa^+^ and urK^+^ concentrations have already been reported by Ribeiro Filho et al. (20) in calves hydrated in continuous flow *via* naso-ruminal route. In the same line, according to Waldrop (22), most of the chloride filtered in the renal glomeruli is passively reabsorbed concurrently to sodium reabsorption. Therefore, the greater excretion of sodium leads to a greater excretion of chloride, justifying the findings of the present study.

Urinary volume progressively increased in animals of all groups. This result reinforces the effect of the tested enteral electrolyte solutions, there was absorption of the solutions resulting in blood volume increase and, consequently, an increase in urine production, as shown in Table 4. Results similar to the present test were also described by Alves et al. (7) in adult cattle and Morais et al. (9) in adult goats.

The blood pH values showed a slight but significant increase (Table 4) in the animals of the ESCaP and ESGly groups, mainly in the ESCaP group at T12h. This finding is possibly due to the composition of the electrolyte solutions. The increase in blood pH was not considered clinically relevant since values remained in the normal range for the species (7.32 to 7.44) and alkalinemia was not noted (17).

During the experimental phase, there was no difference (*P* > 0.05) in the partial pressure of carbon dioxide (pCO_2_) between groups and in the groups over time (Table 4). According to Radostits et al. (23), Carlson and Bruss (17) and Smith (24) the reference values for pCO_2_ in cattle are in the range of 34 to 45 mmHg. When comparing these values to those recorded in the present trial [minimum of 47.3 ± 3.5 and maximum of 51.4 ± 6.4 (Table 4)], pCO_2_ was already above the described reference range at T0h. Similar to the findings from the present study, Dirksen (25) registered values of 35 to 53 mmHg as reference range for pCO_2_ in cattle. Variables such as age, sex, breed, diet and handling of the animals in the present trial may explained the difference in pCO_2_ values in comparison to most of the international literature. Therefore, the confrontation of the present essay data with those from international literature can generate confusion at the time of its interpretation.

Bicarbonate concentrations (HCO${}_{3}^{-}$) showed similar behavior in the three groups (Table 4). It remained practically unchanged at T0h, T3h and T6h, with a slight increase at T9h and T12h (*P* < 0.05). Only the animals in the ESCaP group showed HCO${}_{3}^{-}$ values above the normal range, which according to Carlson and Bruss (17) is between 20 and 30 mmol/L. As with pCO_2_, this increase was mild (33.9 ± 1.7 mmol/L).

The increase in HCO${}_{3}^{-}$ recorded in the animals of the ESCaP group during enteral fluid therapy (T9h and T12h) demonstrates that there was an enhance in the alkaline reserve, which comes from the process of metabolizing the glycemic precursors present in the solutions: propionate, glycerol, and propylene glycol. During the oxidation process of these precursors, which occurs inside the mitochondria to produce glucose, there is a consumption of H^+^ ions causing a gradual process of alkalinization on the organism (26). Therefore, as mentioned by Leal et al. (27), these precursors are also called metabolizable bases.

The base excess is defined as the amount of acid, in mEq/L or mmol/L, necessary to restore the pH of one liter of blood at 37°C and pCO_2_ 40 mmHg, to the value of 7.40 (10). It is considered an independent variable and represents the metabolic component. Negative values below the normal range indicate metabolic acidosis, while positive values above the normal range indicate metabolic alkalosis. No difference in BE was observed between groups (*P* > 0.05). A difference was observed only in ESCaP and ESGly groups throughout the experimental phase (*P* < 0.05). The results obtained in the animals of ESCaP group at T9 (6.7 ± 2.9) and T12h (8.1 ± 1.5) were superior to the reference values (0 to 6 mmol/L) described by Constable (28). These results confirm that ESCaP generates a mild reversible metabolic alkalosis. The mechanism involved in the occurrence of this disorder was the same as described in the discussion of HCO${}_{3}^{-}$. It should be emphasized that metabolic disturbances of this intensity resolve spontaneously, requiring only the suspension of the administration of the electrolyte solution. For this reason, it was called discrete reversible metabolic alkalosis.

The enteral electrolyte solution containing glycerol (ESGly) showed the highest BE values at T6h, but these indices did not exceed the normal range. The tendency toward alkalization demonstrated by this solution is devoid of clinical significance because it did not exceed physiological reference values. ESPrG showed no change in BE during the entire experimental phase (*P* < 0.05) confirming its neutral action on acid-base balance.

Mixed acid-base disorder is characterized by the presence of two or more primary changes in a patient, thereby modifying the expected compensatory responses (29). In the determination of SID, there is an additional concept to the electroneutrality principle, which considers only the determination of strong cations and anions. SID is the sum of strong cation concentrations subtracted from the sum of strong anion concentrations (28, 30). It is used to confirm mixed disturbances, being expressed by the equation: SID = (Na^+^ + K^+^) - (Cl^−^). According to Constable (28), the reference value in cattle is 38–46 mmol/L. Values above the normal range indicate metabolic alkalosis, while values below the normal range indicate metabolic acidosis. The SID values in animals of all groups were not influenced by treatments and time (*P* > 0.05). The indices were similar in all groups (Table 4), confirming that Anion Gap (AG) results and concentrations of strong ions, Na^+^, K^+^, and Cl^−^ in blood remained within the normal range. It reinforces that the composition of the tested enteral solutions in the present trial was adequate.

The kidneys act in the elimination of excess acids and bases of non-respiratory origin playing a fundamental role in the maintenance of acid-base balance. They are considered the third line of defense in this purpose. As shown in Table 4, urinary pH also did not change between groups and within groups throughout the experimental phase (*P* > 0.05). These results show that the tested electrolyte solutions did not cause significant acid-base imbalance. Despite the occurrence of mild reversible metabolic alkalosis in the ESCaP group a renal compensation mechanism was not triggered, proving that the metabolic alkalosis had little effect on the homeostasis of the animals. Although urinary pH measurement is frequently used to assess the acid-base condition of animals when no other analysis is available, Kaneko et al. (31) recommend caution when using this parameter for this purpose. They suggest that there are many factors that can mask the results.

## Conclusions

The enteral electrolyte solutions tested in the present study were effective in expanding blood volume without causing additional fluid and electrolyte imbalances. ESCaP caused mild reversible metabolic alkalosis, therefore it is contraindicated in animals with metabolic alkalosis. Calcium propionate (ESCaP) and propylene glycol (ESPrG) were the precursors with the highest glycemic potential, which makes the use of these solutions suitable in patients with hypoglycemia.

## Data availability statement

The datasets presented in this study can be found in online repositories. The names of the repository/repositories and accession number(s) can be found in the article/supplementary material.

## Ethics statement

The animal study was reviewed and approved by Ethics Committee on the Use of Animals (CEUA) of the Federal University of Viçosa (UFV).

## Author contributions

JR, PE, MA, and RV were responsible for the conception of the study, data analysis, and provided intellectual input on the manuscript. PE, LM, CC, LB, PM, JS, and DE were responsible for carrying out the experimental phase of the study. JR, BC, MdS, and NM were responsible for data interpretation and writing of the manuscript. All authors contributed to the article and approved the submitted version.

## Funding

This study was financed in part by the Coordenação de Aperfeiçoamento de Pessoal de Nível Superior (CAPES) - Brasil.

## Conflict of interest

The authors declare that the research was conducted in the absence of any commercial or financial relationships that could be construed as a potential conflict of interest.

## Publisher's note

All claims expressed in this article are solely those of the authors and do not necessarily represent those of their affiliated organizations, or those of the publisher, the editors and the reviewers. Any product that may be evaluated in this article, or claim that may be made by its manufacturer, is not guaranteed or endorsed by the publisher.
